# Recognizing the mobilization of neutrophils with banded nuclei early after trauma

**DOI:** 10.1111/ijlh.13272

**Published:** 2020-07-06

**Authors:** Pien Hellebrekers, Lilian Hesselink, Albert Huisman, Maarten ten Berg, Leo Koenderman, Luke P.H. Leenen, Falco Hietbrink

**Affiliations:** ^1^ Department of Respiratory Medicine and Laboratory of Translational Immunology University Medical Center Utrecht Utrecht The Netherlands; ^2^ Department of Trauma Surgery University Medical Center Utrecht Utrecht The Netherlands; ^3^ Department of Clinical Chemistry and Hematology University Medical Center Utrecht Utrecht The Netherlands

**Keywords:** banded, diagnosis, infection, neutrophil, trauma

Dear Editors,

After major trauma neutrophil function is negatively affected (eg, diminished responsiveness to stimuli, chemotaxis, phagocytosis^1^), contributing to increased susceptibility of infection. The initial presence of banded, segmented, and hypersegmented neutrophils (based on CD16/CD62L expression/associated with nuclear segmentation) is linked to late infectious complications^2^ and the development of multi‐organ failure^3^ during admission in major trauma patients. Therefore, it is of great importance to recognize neutrophil subsets to obtain all information on neutrophil function after trauma. Automated hematological analyzers made neutrophilic differentiation an easily accessible tool to automatically analyze the number of progenitors and banded cells based on predefined algorithms.^4^

White blood cell counts and, particularly, neutrophil left shifts (increased counts of immature or banded neutrophils) have proven their diagnostic value in bacterial infection.^5^ The value for diagnosis of (sterile) inflammatory diseases is far less obvious. In the case of trauma, the presence of banded neutrophils in the first day has been described.^6^

Despite the consensus regarding the occurrence of banded neutrophils during infection, the situation after traumatic injury is much less clear. Differentiation data obtained with classical microscopy showed fast numbers (10%‐98%) of banded neutrophils recirculating immediately after major trauma.^6^ However, applying automated leukocyte differentiation by a routine hematological analyzer in earlier studies on trauma patients revealed minimal band counts (0%‐5%).^3^ We aimed to investigate the accuracy of a fully automated hematological analyzer to identify the neutrophil left shift following major trauma and compare results to manual differentiation and immunophenotyping by flow cytometry.

Eight samples from six adult polytrauma patients (<48 hours after trauma, injury severity score >16, all males, median age 47 years(range 31‐66y)) and 5 surgical infection patients (female/male, median age 67 years(52‐70), 3 abdominal sepsis, 2 pneumonia) were included in this study. The trauma patients were prospectively included after written informed consent was obtained, as additional flow cytometry analysis was required. The surgical infection patients were retrospectively included after a waiver was provided by the local ethical committee. The study was performed in accordance to the Declaration of Helsinki and approved by the local ethical review committee (METC, protocol number 17‐519/C).

From the trauma cohort, the automated leukocyte differentiation including morphological parameters (proxies for size and lobularity of leucocytes), manual differentiation counts, and expression of CD16/CD62L on neutrophils determined as a proxy of nuclear segmentation^7^ by flow cytometry (see below) were available. From the surgical infection cohort automated and manual differentiation counts of blood leukocytes were obtained by routine diagnostics. In both infection and trauma patients, neutrophil counts were normal or elevated in all cases.

For the automated differentiation, fresh (<1 hour) whole blood samples were measured on the Cell‐Dyn Sapphire hematology analyzer (Abbott Diagnostics). A confidential algorithm from the manufacturer allows the software to distinguish immature, banded, and segmented neutrophils based on light scatter patterns (eg, 0‐degree scatter is a proxy for cell size, and 90‐degree scatter is a proxy for lobularity) with a technique called Multi‐Angle Polarized Scatter Separation (MAPPS). Other neutrophil characteristics could be recognized but were outside the scope of this manuscript. These data are systematically collected with the Utrecht Patient Oriented Database, a relational database infrastructure.^8^

For the manual differentiation, either blood smears of whole blood or cytospins of 1 × 10^5^ leukocytes were used (<4 hours), based on availability (retrospective/prospective inclusion). After initial preparation, the cells were fixed using >99% methanol for 2 minutes and consecutively stained with May‐Grünwald/Giemsa. At least 200 neutrophils were counted per slide. Segmentation was defined as an indentation of ≥2/3 of the width of the nucleus at its widest point of the segment.^9^ Mature neutrophils without indentation were considered banded.^9^

Erythrocytes from fresh whole blood samples were lysed for 15 minutes in ice‐cold NH_4_Cl solution. Leukocytes were washed twice and resuspended in supplemented phosphate‐buffered saline (pasteurized plasma solution (10%), trisodium citrate (0.4%[w/v]))(Sigma‐Aldrich, Merck KgaA, Darmstadt, Germany) to a concentration of 5 × 10^6^ leukocytes. After 30 minutes of incubation with CD16‐Krome Orange (clone 3G8) and CD62L‐ECD (clone DREG56, Beckman Coulter), the cells were washed and analyzed on the Gallios flow cytometer (Beckman Coulter). CD16^dim^/CD62L^high^ neutrophils were considered as “banded” and CD16^high^/CD62L^high^ as “segmented” neutrophils.^7^ Additionally, neutrophils were sorted based on CD16/CD62L expression and additional cytospins were obtained to serve as an internal control. Flow cytometry data were analyzed with FlowJo^®^ v10 software (FlowJo, LLC).

Data are expressed descriptive or as means with standard deviation. Comparisons between different patient or neutrophil subsets groups were made with the paired t test, and normality was tested with D’Agostino's‐Pearson normality test. The Fisher LSD test was used for multiple comparisons. Graphs and tests were conducted using GraphPad Prism 7 (GraphPad Software, Inc) and SPSS (SPSS Inc, IBM). *P*‐values are not adjusted for multiple comparisons. Results were regarded as significant when *P* < .05.

In none of the trauma patient samples, the automated hematology analyzer detected any immature or banded neutrophils (0% ± 0%). In surgical patients with acute infection, the automated differentiation did reveal banded neutrophils (22% ± 8%). In marked contrast to automated analysis, manual differentiation revealed large numbers of banded neutrophils in both trauma patients (37% ± 17%, *P* = .0078) and infection patients (22% ± 6%, *P* = .8125) (Figure 1A,B).

**FIGURE 1:**
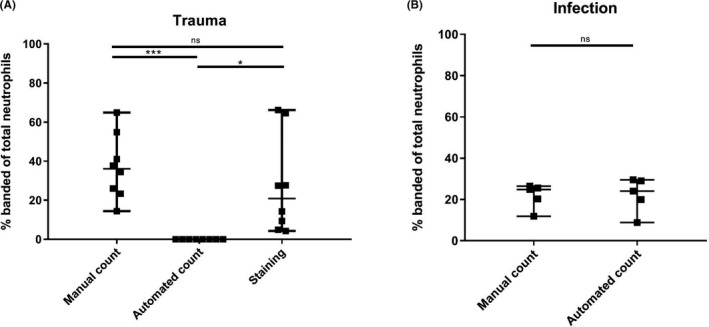
Band cell counts in trauma patients (A) and infection patients (B) as a percentage of total neutrophils. In trauma (A) automated neutrophil differentiation did not detect any banded neutrophils, whereas manual counts (*P* = .0001) and CD16‐staining did. No significance difference was found between manual counts and CD16^dim^ band counts

Furthermore, CD16/CD62L staining revealed fast amounts of CD16^dim^ neutrophils (Figure 2A + B) varying between 10% and 60%. Sorting of CD16^dim^/CD62L^high^ and CD16^high^/CD62L^high^ neutrophils demonstrated a great enrichment of banded neutrophils in the CD16^dim^ gate (Figure 2C). There was no significant difference in band counts in trauma between manual differentiation (37% ± 17%) and CD16^dim^ counts (37% ± 25%, *P* = .2687). Although the means are very similar, between individual samples, the banded count between manual differentiation and CD16^dim^ did differ sometimes on an individual level.

**FIGURE 2:**
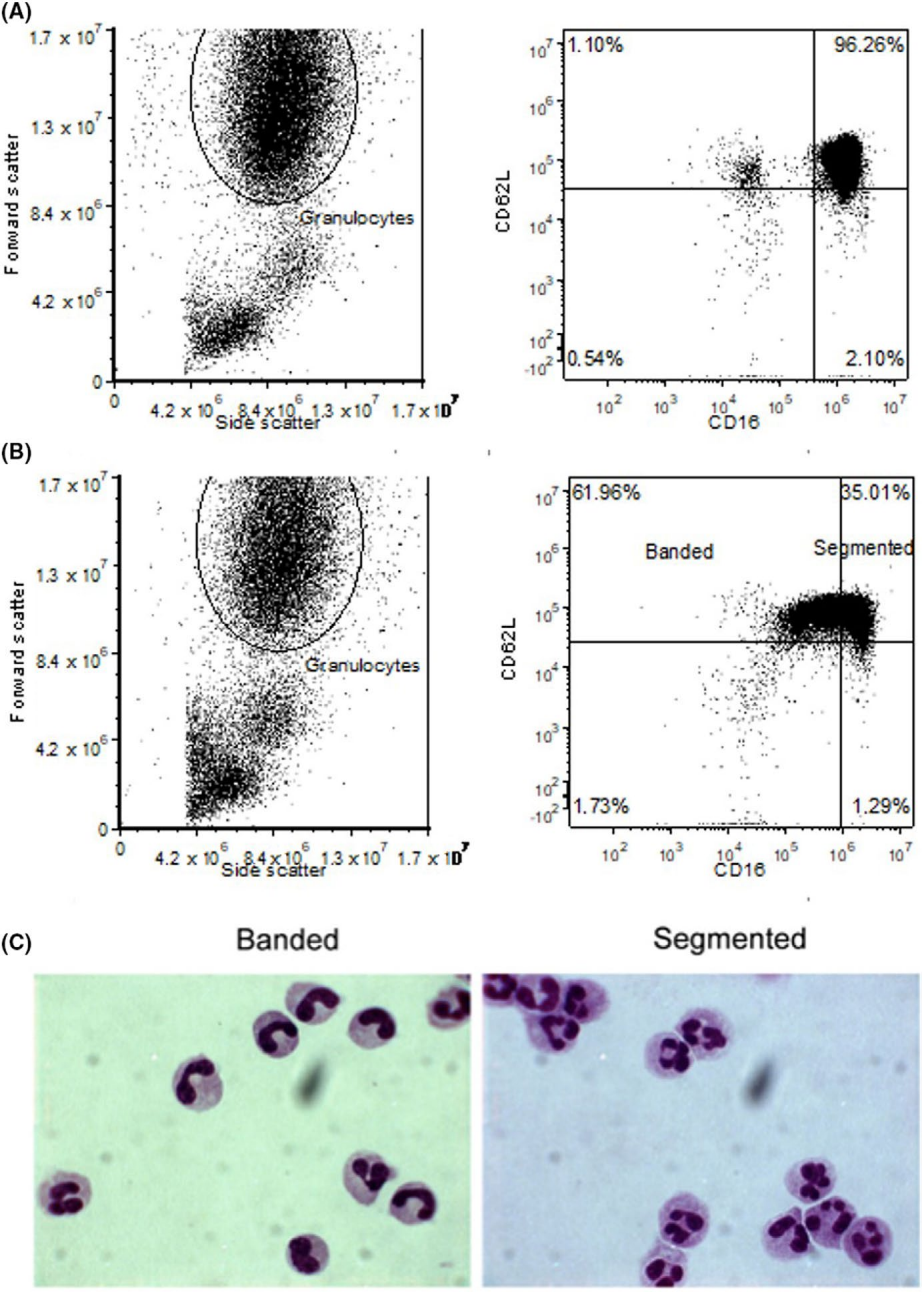
A and B, Gating strategy for flow cytometry identification of immature and banded neutrophils. In A an example of a healthy control, in B an example of a polytrauma patient upon presentation at the emergency department. Granulocytes are identified on their specific forward/side scatter, after which neutrophils are characterized on CD16/CD62L expression. For sorting, Q1 was identified as the banded and Q2 as mature neutrophil gate. See also Figure 2B. C. Sorting of CD16^dim^ and CD16^high^ neutrophils classifies banded and segmented neutrophils correctly. CD16^dim^ and CD16^high^ neutrophils were sorted and single‐cell cytospins prepared and scored for the presence of banded cells. Banded cells were enriched in the CD16^dim^ gate

To further explore this difference in banded neutrophils by different techniques, we analyzed scatter readings used in the algorithm. Based on 0*°* light scattering, infection patients had a significantly higher neutrophil cell size than trauma patients (166 ± 7.83 arbitrary units [AU] vs.151 ± 6.72 AU; *P* = .011). Neutrophil cell size from infection patients samples was also higher than reference values, whereas trauma patient samples remained within the upper limits of normal reference values (120‐159 AU). Neutrophil lobularity (90*°* light scattering) remained within reference values in both groups without significant differences between groups. In the trauma group, no significant differences in cell size between CD16^dim^ and CD16^high^ neutrophils were found. An exemplary scatter plot is shown in Figure 3.

**FIGURE 3:**
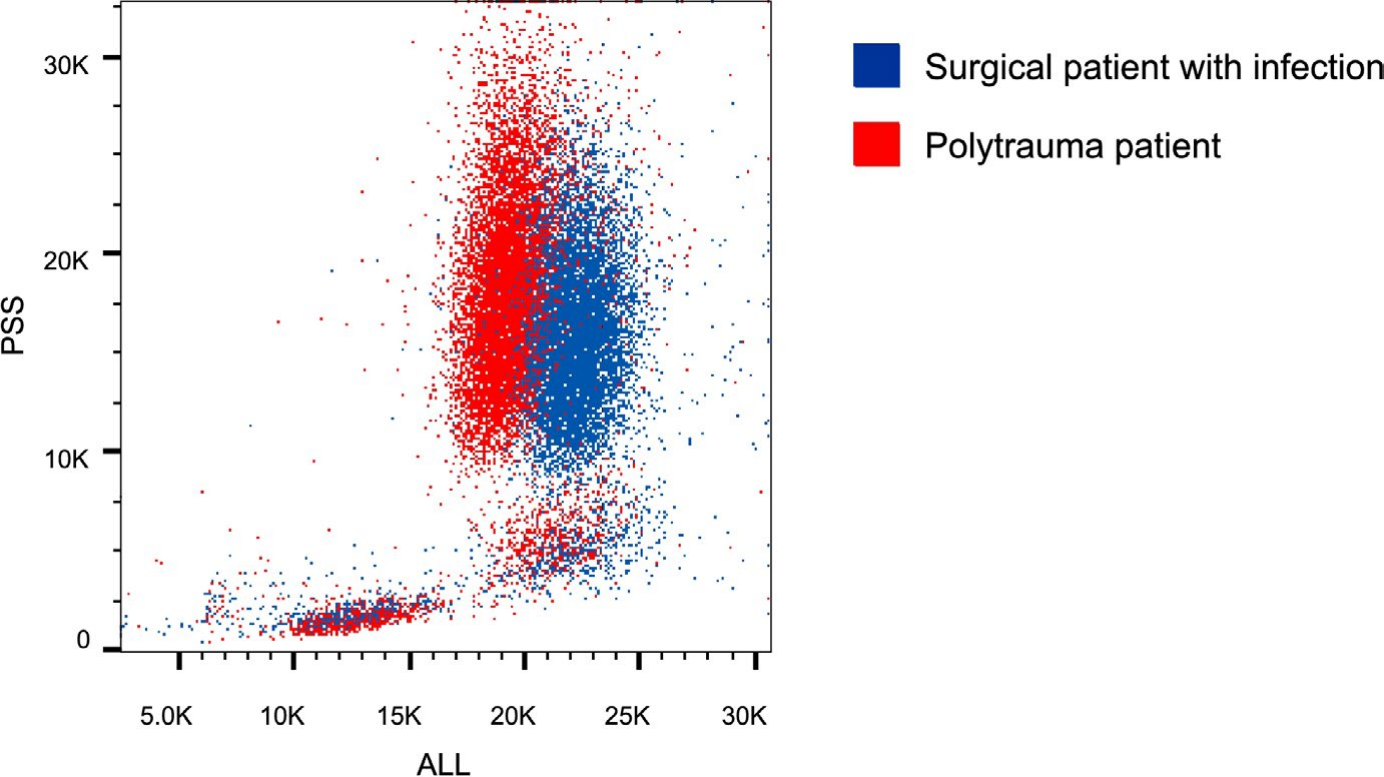
Exemplary scatter plots showing forward scatter (size, ALL) and side scatter (lobularity, PSS) of infection and trauma patients

The Cell‐Dyn algorithm uses neutrophil scatter parameters including increased 0‐degree scatter (proxy for neutrophil size) and 90‐degree scatter (proxy for neutrophil lobularity) to detect immature neutrophils (exact algorithm remains confidential). Thus, cell size is part of this algorithm and we found that neutrophil cell size was increased during infection, whereas it was not after trauma. An increase in neutrophil size has been found after in vitro activation with inflammatory mediators.^10^ So, it is possible that mean neutrophil size was not increased because of the presence of larger banded neutrophils, but simply because of neutrophil enlargement due to in vivo activation of neutrophil during infections. This is supported by our finding that there was no difference in cell size between banded (CD16^dim^/CD62L^high^) and segmented (CD16^high^/CD62L^high^) neutrophils after trauma. This might be (part of) the explanation for the inability of the hematological analyzers algorithm to detect these banded cells. For the purpose of this study, only one analyzer was tested. Although it is tempting to speculate that other analyzers would have the same problem, as algorithms are mostly based on light scatter patterns, caution should be taken in extrapolating these findings to other hematological analyzers.

Although we tested a limited number of patients, we provided proof for the principle that algorithm‐based automated neutrophil differentiation using a Cell Dyn hematology analyzer is not suited to detect the presence of banded neutrophils in the circulation following major trauma. On the contrary, it accurately detected banded neutrophils in patients suffering from infections. Phenotyping neutrophils based on their CD16/CD62L expression was a more accurate method to automatically identify the presence of banded neutrophils in the circulation of trauma patients. Thereafter, studies should focus on the differences in banded neutrophils and whether these cells only differ in phenotype or also in function.

## CONFLICT OF INTEREST

The authors have no competing interests.

## AUTHORS’ CONTRIBUTION

PH and LH were responsible for data collections, analysis, and drafting of the manuscript. AH and MtB were responsible for data collections and critical review of the manuscript. LK, LL, and FH were responsible for data analysis and critical review of the manuscript. All authors read and approved the final version.

## References

[1] Hazeldine J , Hampson P , Lord JM . The impact of trauma on neutrophil function. Injury. 2014;45(12):1824‐1833. 25106876 10.1016/j.injury.2014.06.021

[2] Leliefeld PHC , Pillay J , Vrisekoop N , et al. Differential antibacterial control by neutrophil subsets. Blood Adv. 2018;2(11):1344‐1355. 29895625 10.1182/bloodadvances.2017015578 PMC5998927

[3] Botha AJ , Moore FA , Moore EE , Sauaia A , Peterson VM . Early neutrophil sequestration after injury: A pathogenic mechanism for multiple organ failure. J Trauma. 1995;39(3):411‐417. 7473901 10.1097/00005373-199509000-00003

[4] Chabot‐Richards DS , George TI . White blood cell counts: Reference methodology. Clin Lab Med. 2015;35(1):11‐12. 25676369 10.1016/j.cll.2014.10.007

[5] Nahm CH , Choi JW , Lee L . Delta neutrophil index in automated immature granulocyte counts for assessing disease severity of patients with sepsis. Ann Clin Lab Sci. 2008;38(3):241‐246. 18715852

[6] Köller M , Wick M , Muhr G . Decreased leukotriene release from neutrophils after severe trauma: Role of immature cells. Inflammation. 2001;25(1):53‐59. 11293666 10.1023/a:1007027712387

[7] Pillay J , Ramaker BP , Kamp VM , et al. Functional heterogeneity and differential priming of circulating neutrophils in human experimental endotoxemia. J Leukoc Biol. 2010;88(1):211‐220. 20400675 10.1189/jlb.1209793

[8] Ten Berg MJ , Huisman A , van den Bremt PM , Schobben AF , Egberts AC , van Solinge WW . Linking Laboratory and medication data: new opportunities for pharmacoepidemiological research. Clin Chem Lab Med. 2007;45(1):13‐19. 17243908 10.1515/CCLM.2007.009

[9] Zini G , Bain B , Bettelheim P , et al. A European consensus report on blood cell identification: terminology utilized and morphological diagnosis concordance among 28 experts from 17 countries within the European LeukemiaNet network WP10, on behalf of the ELN morphology faculty. Br J Haematol. 2010;151(4):359‐364. 20812999 10.1111/j.1365-2141.2010.08366.x

[10] Hesselink L , Heeres M , Paraschiakos F , et al. A rise in neutrophil cell size precedes organ dysfunction after trauma. SHOCK. 2019;51(4):439‐446. 29889813 10.1097/SHK.0000000000001200

